# Intravenous Thrombolysis for Acute Ischemic Stroke during the COVID-19 Pandemic—Polish Single-Center Retrospective Cohort Study

**DOI:** 10.3390/life12071068

**Published:** 2022-07-17

**Authors:** Tomasz Chmiela, Michalina Rzepka, Maciej Kuca, Karolina Serwońska, Maciej Laskowski, Agnieszka Koperczak, Joanna Siuda

**Affiliations:** 1 Department of Neurology, Faculty of Medical Sciences in Katowice, Medical University of Silesia, 40-752 Katowice, Poland; michalina.rzepka93@gmail.com (M.R.); jsiuda@sum.edu.pl (J.S.); 2 Students’ Scientific Association, Department of Neurology, Faculty of Medical Sciences in Katowice, Medical University of Silesia, 40-752 Katowice, Poland; maciej.kuca99@gmail.com (M.K.); kserwonska@gmail.com (K.S.); maciek.lask@gmail.com (M.L.); koperczak99@gmail.com (A.K.)

**Keywords:** ischemic stroke, thrombolytic treatment, COVID-19, risk factors

## Abstract

COVID-19 has affected the entire world and has had a great impact on healthcare, influencing the treatment of patients with acute ischemic stroke (AIS). The aim of this study was to determine the impact of the COVID-19 pandemic on the care of patients with AIS. We performed a retrospective analysis of 1599 patients diagnosed with AIS and hospitalized in the authors’ institution from January 2018 to December 2021. The final sample consisted of 265 patients treated with thrombolysis without a diagnosis of COVID-19. The initiation of thrombolytic treatment during the pandemic was delayed (2:42 ± 0:51 vs. 2:25 ± 0:53; *p* = 0.0006). The delay was mainly related to the pre-hospital phase (1:41 ± 0:48 vs. 1:26 ± 0:49; *p* = 0.0014), and the door-to-needle time was not affected. There were no differences in stroke severity and patients’ outcomes. Patients with AIS were less likely to have previously been diagnosed with atrial fibrillation (16.9% vs. 26.7%; *p* = 0.0383), ischemic heart disease (25.3% vs. 46.5%; *p* = 0.0003) and hyperlipidemia (31.2% vs. 46.5%; *p* = 0.0264). Patients treated during the pandemic had higher glycemia (149.45 ± 54. vs. 143.25 ± 60.71 mg/dL; *p*= 0.0012), while no significant differences in their lipid profiles were found. Conclusions: The COVID-19 pandemic affected the treatment of AIS patients locally at our stroke center. It caused treatment delay and hindered the recognition of risk factors prior to the occurrence of AIS.

## 1. Introduction

Severe acute respiratory syndrome coronavirus 2 (SARS-CoV2), which emerged in China in 2019, causes acute respiratory disease. COVID-19 has posed a great challenge to the entire world and has reached the scale of a global pandemic, changing the lives of millions of people [1]. By May 2022, COVID-19 had affected 515 million people worldwide and accounted for 6.2 million deaths [2]. The necessity to face this new threat has directly influenced the way healthcare systems currently operate.

During the COVID-19 pandemic, new challenges emerged in the care of patients with acute ischemic stroke (AIS). Various studies suggested that AIS patients that were infected with SARS-CoV2 in some ways differed from the cases that could not be attributed to COVID-19 [3,4,5,6]. Therefore, COVID-19 is a factor that needs to be considered when approaching patients showing symptoms of AIS. AIS patients with COVID-19 were younger, more men were affected than women, patients were more likely to have a large-vessel occlusion and the detection of the stroke was delayed [3,4,5]. The study by Vogrig (2021) suggested that these patients were also prone to having severe neurological deficits at presentation and multiple vascular territories involved [5]. The pathomechanism in which SARS-CoV2 contributes to stroke still remains unclear [1]. The virus enters the cells through the angiotensin-2 receptor, which activates the renin–angiotensin axis and reduces the expression of ACE2 while increasing the levels of angiotensin II [7] and decreasing the levels of angiotensin 1–7 (which is now considered to be the key antithrombotic and anti-inflammatory protein). SARS-CoV2 infection may also lead to oxidative stress damage, endothelial dysfunction, the activation of the von Willebrand factor and a dysregulated immune response [1].

The pandemic of coronavirus disease (COVID-19) has had a great impact on stroke healthcare, indirectly influencing the outcome of AIS patients. Hospitals have been overloaded since the beginning of the pandemic, and as a result, AIS patients sometimes received delayed treatment, which threatened their lives [8]. Koge’s (2021) research suggested that the time from hospital arrival to imaging and to thrombolysis was prolonged compared with the pre-COVID-19 period [9]. A study from China showed a reduction in performed thrombolysis of over 25% after COVID-19 outbreaks [10]. Multiple studies suggested a decrease in stroke diagnoses during the pandemic [11,12]. One of the potential reasons for this was a fear of becoming infected in the hospital. This may have caused patients with milder symptoms to stay at home. Additionally, the isolation of the elderly from their family members may have resulted in a significant delay in them seeking medical attention. Furthermore, a significant increase in emergency calls may have led to an improper activation of the stroke protocol [12]. Patients with SARS-CoV2 infection frequently developed respiratory symptoms before AIS manifestations, which could have caused misdiagnoses or delayed stroke diagnoses [5,12].

Since the beginning of the pandemic, the world has been trying to adapt new strategies to minimize the impact of the pandemic on public health. It is worth noting that the COVID-19 pandemic poses a threat in many areas, such as in health and economics. A country’s COVID-19 risk level is dependent on their development level, economy and infrastructure. Access to healthcare and socioeconomic vulnerability should be considered to estimate the level of threat associated with the COVID-19 pandemic. Understanding this risk factor and the development of estimators is crucial in determining strategies for specific countries. [13,14] The aim of this study is to evaluate the impact of the COVID-19 pandemic on the care of patients with AIS treated with thrombolysis, taking into account the vascular risk factors, and the treatment of AIS, with a particular emphasis on the acute phase of the disease, as well as to analyze the secondary prevention in this group of patients. Unlike most studies on AIS care during the COVID-19 pandemic, this paper is focused on the indirect impact of the pandemic on the treatment of AIS patients without SARS-CoV-2 infection. We would like to draw the reader’s attention to the quality of primary prevention during the COVID-19 pandemic, as neglecting this area may have long-term negative consequences on public health.

## 2. Materials and Methods

**Figure 1:**
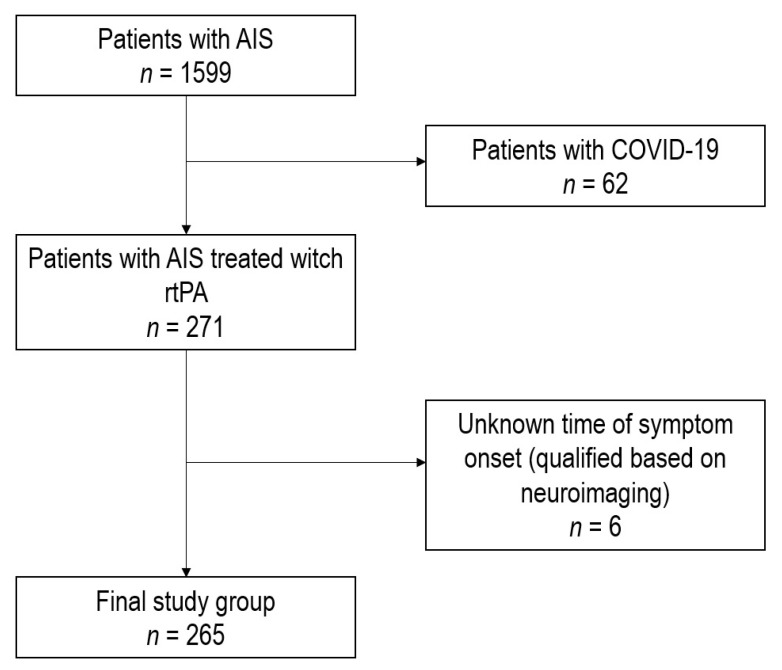
Final group creation process. AIS—acute ischemic stroke; rtPA—recombinant tissue plasminogen activator.

We performed a retrospective analysis of all patients diagnosed with AIS admitted to the Central Clinical Hospital of the Medical University of Silesia in Katowice, a tertiary stroke center, from January 2018 to December 2021. Patients admitted from March 2020 to December 2021 were included in the group of patients treated during the COVID-19 pandemic. Patients admitted before March 2020 were included in the group of patients treated before the COVID-19 pandemic. The initial group consisted of 1599 individuals. Due to the very small group of patients diagnosed with COVID-19 having been treated with thrombolytic therapy (*n* = 6), which was not suitable for statistical analysis, and the aim of the study, which was to assess the indirect impact of the pandemic on patients with AIS, patients diagnosed with COVID-19 at admission were excluded from the study (*n* = 62). The analysis concerned patients treated with thrombolysis; patients in whom the time of symptom onset could not be determined were excluded from the study (e.g., patients that qualified for thrombolysis based on neuroimaging, *n* = 6). The process of the final group creation is presented in Figure 1. 

The final group consisted of 265 patients (16.6%)—135 females (50.9%) and 130 males (49.1%)—aged 71.18 ± 12.67 years (mean ± SD). In the final group, clinical data regarding the National Institutes of Health Stroke Scale (NIHSS) score, as well as the Modified Rankin Scale (mRS) score, at admission and discharge were gathered. Data on the course of thrombolytic treatment, stroke location, secondary prevention, risk factors and bloodwork results were also collected.

The statistical analysis was performed with Statistica 13.3 (TIBCO Software Inc., Palo Alto, CA, USA (2017) and Statistica (data analysis software system, Tulsa, OK, USA, version 13. http://statistica.io). The quantitative variables are presented as the arithmetic mean and standard deviation (normally distributed variables) or the median and interquartile range (variables of abnormal/skewed distribution). The normality of distribution was assessed with the Shapiro–Wilk test. Qualitative variables are presented as absolute values and percentages.

The intergroup differences for the quantitative variables were assessed with an analysis of variance (normally distributed variables) or the Mann–Whitney U test (variables of skewed distribution). Fisher’s exact test or the chi-square test were performed for qualitative variables. The relationships of the quantitative variables were assessed with Spearman’s rank correlation coefficient. Statistical significance was established at *p* < 0.05.

Due to the retrospective nature of the work and data anonymization, the Ethics Committee of the Medical University of Silesia waived the requirement to obtain ethical approval for this study.

## 3. Results

### 3.1. Demographic Data and Stroke-Risk Factors Diagnosed before Admission—Impact on COVID-19 Pandemic

There were no differences in age and sex between the groups of patients treated before and during the COVID-19 pandemic. Patients admitted to the hospital during the pandemic were less likely to have previously been diagnosed with atrial fibrillation (26.7 vs. 16.9%; *p* = 0.0383), ischemic heart disease (46.5 vs. 25.3%; *p* = 0.0003), valvular disease (27.7 vs. 15.6%; *p* = 0.0144), hyperlipidemia (46.5 vs. 31.2%; *p* = 0.0264) and carotid artery narrowing (20.8 vs. 6.5%; *p* = 0.0006). There was no difference in the prevalence of hypertension or diabetes mellitus. Detailed data are provided in Table 1.

### 3.2. The Course of Thrombolytic Treatment and the COVID-19 Pandemic

Patients treated during the COVID-19 pandemic were admitted to the hospital later after the onset of symptoms than before the pandemic (on average: 1 h 41 min vs. 1 h 26 min; *p* = 0.0014). This resulted in a significantly later initiation of thrombolytic treatment in this group of patients (2 h 42 min vs. 2 h 25 min; *p* = 0.0014). No differences were found in in-hospital times, i.e., door-to-imaging and door-to-needle. Patients admitted during the pandemic were more likely to have had a prior disability—mRS (interquartile range 0–0 vs. interquartile range 0–2; *p* = 0.0004). There were no differences in stroke severity, discharge performance, iatrogenic hemorrhages and in-hospital mortality. Detailed data are provided in Table 2.

### 3.3. Diagnostic Workup, Secondary Prevention and COVID-19 PANDEMIC

During diagnosis in the stroke unit, there was no difference in atrial fibrillation diagnosed de novo. There were no significant differences concerning LDL cholesterol, total cholesterol or triglycerides. However, there was a significantly higher HDL level in patients treated during the pandemic (47.23 vs. 51.99 mg/dL; *p* = 0.0011). There were no differences concerning diabetes prevalence; nevertheless, patients treated during the pandemic had a significantly higher glucose level at admission. There was no difference in the prevalence of hypertension. There were no differences in the frequency of valvular disease found based on echocardiography and the frequency of hemodynamically significant carotid stenosis found on angio-CT.

There were no differences in the frequency of prescribing antiplatelet, anticoagulant or vascular surgery referrals recommended after discharge from the hospital. Detailed data are provided in Table 3.

## 4. Discussion

In this study, we reviewed the documentation of stroke patients treated with thrombolytic therapy to evaluate any changes in ischemic stroke management and reperfusion outcome due to the COVID-19 pandemic. We observed no significant differences in age and gender between the groups of patients in pre- and COVID-19 periods, while other studies were inconclusive [4,13,14].

Contrary to other studies that suggested a decrease in stroke diagnoses during the pandemic [11,12,15], in the authors’ institution, an increase in the frequency of hospital admissions in patients with AIS was observed. Before the pandemic, an average of 29.9 patients per month (mean from 01.2018 to 03.2020) and 35.8 patients per month during the pandemic, were hospitalized. This is most likely due to the reduction in the number of centers providing care to patients with AIS due to the creation of hospitals dedicated to treating patients with COVID-19 at the expense of previously operating neurological departments. We also observed the more frequent referral of patients from more distant locations due to the temporary closure of other neurological departments. Throughout the pandemic period, patients without SARS CoV-2 infection were admitted to our institution. For only a few months, within the structure of our hospital, was there a department for COVID-19-positive patients, which explains the relatively low number of COVID-19 patients in this study. We believe that these organizational changes are most likely responsible for the increased frequency of the hospitalization of AIS patients in our center. We also did not observe a reduction in the number of thrombolyses performed in our center, which was reported in other studies [11,12,15]. As mentioned earlier, the local situation of our center explains the observed differences with the results of global studies.

There are literature data suggesting that stroke patients hospitalized during the COVID-19 pandemic were younger and men were more frequently affected than women in comparison to the pre COVID-19 period [4,16]. In our study, patients diagnosed with COVID-19 were excluded from the analysis, which might have resulted in the demography data being consistent with the pre-COVID-19 period.

In our research, we observed that patients seemed to have fewer cardiovascular risk factors, such as atrial fibrillation, hyperlipidemia, hyperglycemia, ischemic heart disease or valvular disease. However, there was no difference in atrial fibrillation diagnosed de novo after a stroke. In most studies that compared pre-COVID-19 and COVID-19 periods, hyperlipidemia was one of the risk factors most commonly undiagnosed [8,16]. In our study, stroke patients had rarely been diagnosed with hyperlipidemia before AIS; however, they did not differ significantly in the objective laboratory assessment or in the frequency of lipid-lowering treatment recommendations, which may suggest the underdiagnosis of lipid metabolism disorders. Underestimation of the frequency of risk factors diagnosed before AIS is most likely linked with the lack of regular health control and the decline of routine health services as well as the poor access to healthcare facilities during the pandemic. Strict lockdown measures and public anxiety might have resulted in patients not seeking medical care when necessary. It is worth noting that patients with AIS treated during the COVID-19 pandemic had comparable lipid profiles and higher average blood glucose, which may suggest that the actual prevalence of vascular risk factors may be underestimated. The decrease in the number of atrial fibrillation and ischemic heart disease diagnoses during the pandemic was also previously reported in the literature [8]. However, the number of atrial fibrillation diagnoses de novo is consistent with the pre-COVID-19 period, which may suggest that the recognition of atrial fibrillation in our center was similar to the period before the pandemic. The data from our research regarding undiagnosed cardiological and endovascular problems such as valvular disease and carotid artery stenosis are another concern that should be addressed in the future. It is worth noting that in our study, the frequency of both valvular disease and significant carotid artery stenosis measured in objective examinations—echocardiography and angio-CT, respectively—was similar in pre- and COVID-19 periods.

Our study found no differences in the recommended secondary prevention in patients with AIS without COVID-19 compared to the pre-pandemic period.

The increasing number of studies indicated that during the pandemic AIS patients were treated with delay [8,16,17,18,19,20]. In our research, the period between stroke onset and hospital arrival time was most commonly affected, resulting in a prolongation of onset-to-needle time of 17 min (*p* = 0.0061). However, there was no significant change in door-to-needle time. Reperfusion therapies and stroke admissions were maintained during the pandemic. This delay could be related to many different factors, some of which are infection prevention, precautions in the emergency department, a lack of transportation, and new protocols for patient triage due to the pandemic [10,21]. The delay in thrombolytic therapy has been the subject of several studies that demonstrated possible effects of the pandemic on every stage of acute stroke treatment. Some publications indicated that the stroke onset-to-door time was prolonged [16,20], while others suggested that the door-to-needle time was longer than in the pre-COVID-19 period [17]. There were also publications that divided door-to-needle time into prolonged door-to-CT [19] and CT-to-needle [8]. The study by Roushdy et al. suggested that due to the shorter transportation time and availability of caregivers, the onset-to-door time could even possibly be shorter than in the pre-pandemic period [22]. In the case of our center, more frequent referrals of patients from more distant locations were observed, which could have prolonged the onset-to-door time. This was most likely related to the reduction in the number of centers and frequent temporary restrictions on admissions in other institutions. In the case of our institution, the restrictions related to COVID-19 did not affect the door-to-needle time as the procedures related to qualification for reperfusion treatment were carried out independently within the emergency room structures. Thus, the waiting time for COVID-19 test results did not affect the door-to-needle time. The differences concerning various studies depicting the delay suggest that there is no single cause responsible for the prolongation in thrombolytic treatment; therefore, finding a universal solution to this problem is unlikely.

In our study, patients admitted to the hospital during the pandemic were more likely to have a prior disability, which was shown by a higher initial mRS compared to pre-pandemic patients. However, there was no difference in the stroke severity and the treatment efficiency (there was no significant change in the NIHSS score on admission and on discharge, and no significant change in the mRS on discharge and the mortality rate compared with pre-COVID-19 periods). Existing studies suggested that not only the severity of stroke but also the outcome was much worse in the COVID-19 period [4,5,22,23]. The difference between our research and previous publications concerns the type of patients that were under evaluation. These studies mostly analyzed all patients with stroke that were admitted to the hospital regardless of whether they had received thrombolytic therapy or were infected with SARS-CoV2. Therefore, it is difficult to compare the data from our study in which the delay related to the pandemic could have resulted in the ineligibility for reperfusion treatment with other studies. Nevertheless, there was no change in the thrombolysis rate at the authors’ institution. Studies that directly compared non-COVID-19 with COVID-19 cases indicated that COVID-19 as a risk factor significantly increases both stroke severity and the mortality rate [4,5] (which was particularly high in situations when a stroke occurred in conjunction with severe respiratory disease requiring ICU hospitalization) [24]. It is also worth mentioning that the pandemic time period was associated with increased stress levels and depression episodes, which could also be associated with more severe strokes [8,25,26].

Our study has limitations that should be considered. First, because of the retrospective single-center nature of our study, selection and sampling might have been biased. Therefore, our observations should be interpreted with caution. Due to the local situation during the COVID-19 pandemic, our institution was one of the few hospitals that admitted AIS patients without SARS-CoV2 infection. Second, only patients treated with thrombolysis were included in this study, which may prevent the generalization of the results to all patients with AIS. The single-center nature of the study and the fact that only patients treated with thrombolysis were included in the analysis resulted in a relatively small sample size, with limited external validity regarding this study. Third, only patients without a diagnosis of COVID-19 were analyzed, so the study did not evaluate the direct impact of SARS-CoV-2 infection on the course of AIS.

## 5. Conclusions

Our study demonstrated that the COVID-19 pandemic affected the management of AIS patients at our tertiary stroke center. In particular, the evolving pandemic has resulted in the poorer recognition of cardiovascular risk factors. A tendency towards disregarding this important aspect of cardiovascular health may have a long-term impact on public health. Moreover, the pandemic has led to delayed reperfusion treatment in AIS patients without COVID-19 at our institution in Poland. However, the external validity of and possible geographic variations in our observations require further study.

## Figures and Tables

**Table 1:** Group comparison between individuals with AIS treated with rtPA hospitalized before and during the COVID-19 pandemic, regarding demographic and risk factors. The Mann–Whitney U test was performed for quantitative variables, and Fisher’s exact test was performed for qualitative variables.

KERRYPNX	Before COVID-19 Pandemic	During COVID-19 Pandemic	*p*
Gender *n* (%)			0.2855
Female	49 (48.5)	86 (55.8)	
Male	52 (51.5)	68 (44.2)	
Age (years)	70.6 ± 14.3	71.8 ± 11.1	0.7429
Hypertension *n* (%)	82 (81.2)	120 (77.9)	0.6618
Hyperlipidemia *n* (%)	47 (46.5)	48 (31.2)	0.0264
Atrial fibrillation *n* (%)	27 (26.7)	26 (16.9)	0.0383
Atrial fibrillation de novo *n* (%)	13 (12.9)	18 (11.7)	0.4610
Diabetes mellitus *n* (%)	28 (27.7)	38 (24.7)	0.3768
History of myocardial infarction *n* (%)	20 (19.8)	13 (8.4)	0.0069
Ischemic heart disease *n* (%)	47 (46.5)	39 (25.3)	0.0003
Valvular disease *n* (%)	28 (27.7)	24 (15.6)	0.0144
Significant carotid artery stenosis *n* (%)	21 (20.8)	10 (6.5)	0.0006
History of acute ischemic stroke *n* (%)	20 (19.8)	31 (20.1)	0.5148

**Table 2:** Group comparison between individuals with AIS treated with rtPA and hospitalized before and during the COVID-19 pandemic, regarding the course of thrombolytic treatment. The Mann–Whitney U test was performed for quantitative variables, and Fisher’s exact test was performed for qualitative variables. CT—computed tomography; NIHSS—the National Institutes of Health Stroke Scale; mRS—Modified Rankin Scale; IQR—interquartile range.

	Before COVID-19 Pandemic	During COVID-19 Pandemic	*p*
Treatment times M (SD)			
Onset-to-door (h)	1:26 ± 0:49	1:41 ± 0:48	0.0014
Door-to-CT (h)	0:26 ± 0:12	0:30 ± 0:21	0.1912
Door-to-needle (h)	1:00 ± 0:23	0:59 ± 0:27	0.4649
Onset-to-needle (h)	2:25 ± 0:53	2:42 ± 0:51	0.0006
Median NIHSS (points) (IQR)			
Admission	8 [4–12]	8 [4–12]	0.8292
Discharge	4 [0–8]	3 [0–9]	0.8729
∆ NIHSS	3 ± [0–4]	3 [0–4]	0.8658
Median mRS (points) (IQR)			
Admission	0 [0–0]	0 [0–2]	0.0004
Discharge	3 [1–4]	2 [0–4]	0.7717
Days in hospital (days)	9 [8–12]	9 [8–11]	0.3563
Thrombectomy *n* (%)	19 (18.8)	37 (24.0)	0.2793
Hemorrhagic transformation of stroke *n* (%)	11 (10.9)	24 (15.6)	0.1820
In hospital death *n* (%)	18 (17.8)	27 (17.5)	0.5448

**Table 3:** Group comparison between individuals with AIS treated with rtPA and hospitalized before and during the COVID-19 pandemic, regarding laboratory results, artery stenosis and secondary prevention. The Mann–Whitney U test was performed for quantitative variables, and Fisher’s exact test was performed for qualitative variables. LDL—low-density lipoprotein; HDL—high-density lipoprotein; CRP—C-reactive protein; NOAC—novel oral anticoagulants; LMWH—low-molecular-weight heparin; VKA—vitamin K antagonists; IQR—interquartile range.

	Before COVID-19 Pandemic	During COVID-19 Pandemic	*p*
Laboratory results Median (IQR)			
LDL mg/dL	107 [77–137]	103 [76–130]	0.6382
HDL mg/dL	44.5 [36–53]	51.5 [43–60]	0.0012
Total cholesterol mg/dL	178 [140–216]	178 [143.5–212.5]	0.9919
Triglycerides mg/dL	112.5 [69.5–145.5]	105 [68–142]	0.1879
Glycemia mg/dL	123 [99–146]	135 [108–162]	0.0357
CRP mg/L	3.45 [0–6.55]	3.8 [0–9.3]	0.5811
Troponin ng/L	13.8 [6.1–21.4]	[14.6–23.0]	0.9077
Artery stenosis *n* (%) (angio-CT)	23 (20.8)	30 (18.3)	0.6543
Valvular disease *n* (%) echocardiography	31 (30.7)	46 (28.0)	0.8063
Secondary prevention *n* (%) Antiplatelet therapy: Single Dual	55 (54.5) 8 (0.8)	81 (49.7) 9 (0.6)	0.6697
Anticoagulation *n* (%):			0.2283
NOAC	18 (17.8)	25 (16.2)	
Rivaroxaban	5 (5.0)	8 (5.2)	
Apixaban	7 (6.9)	15 (9.7)	
Dabigatran	5 (5.0)	5 (3.2)	
LMWH	10 (9.9)	21 (13.6)	
VKA	4 (4.0)	1 (0.6)	
Statins	82 (81.8)	131 (79.9)	0.8657

## Data Availability

The data presented in this study are available from the corresponding author upon request.
